# Comparison of human milk bottle with infant/toddler test weights in the community setting

**DOI:** 10.1186/s40795-024-00911-4

**Published:** 2024-07-25

**Authors:** Anita L. Esquerra-Zwiers, Madeline Heter, Anastasia Perecki, Olivia Jackson, D. Addam Jongekryg, Brian Yurk

**Affiliations:** 1 Hope College Nursing Department, Holland, MI USA; 2 Hope College Nursing Department, Corewell Health William Beaumont University Hospital, Royal Oak, MI USA; 3 Hope College Nursing Department, Children’s Hospital of Michigan, Detroit, MI USA; 4 Hope College Nursing Department, DeVos Children’s Hospital, Grand Rapids, MI USA; 5 Hope College Nursing Department, Medical University of South Carolina, Charleston, SC USA; 6 Hope College Mathematics and Statistics Department, Holland, MI USA

**Keywords:** Test weights, Infant, Newborn, Milk, Human

## Abstract

**Background:**

The accuracy of infant intake using test weights (TWs), the change in weight before and after an infant feeds, has only been validated in hospitalized premature infants. This study’s primary aim was to identify how accurate parent infants/toddler (< 2 years old) TWs are at measuring infant intake.

**Methods:**

Data were collected from 101 paired bottle and infant/toddler TWs with 31 participants. Parents participated in the feeding sessions by completing infant/toddler TWs blinded to the researcher. Research assistants completed human milk bottle TWs. Infants were fed previously expressed human milk, initially 30 g, but volumes were increased to not exceed the scale’s capacity.

**Results:**

The mean difference between the bottle TWs measured using the Tanita and OHAUS scales was not significantly different from zero (95% CI (Tanita – OHAUS): (-0.251, 0.108) g). The mean difference between infant/toddler and bottle TWs was significantly different from 0 (95% CI (infant—bottle): (-3.45, -0.915 g or -3.57, -0.95 mL). Infant/toddler and bottle TWs were in agreement with a difference of 2.18 g (SD = 6.63) or 2.25 mL within the scale stated accuracy.

**Conclusions:**

The Tanita infant digital scale accurately measures bottle TWs. The differences in parent infant/toddler TWs are within a clinically acceptable range.

## Introduction

Although human milk is the preferred nutrition for all infants and is recommended by multiple organizations as the exclusive infant nutrition for up to 2 years of age [1, 2], less than 25% of US mothers [3] and less than 44% of mothers globally are meeting this goal [4]. A primary reason for discontinuation is perceived insufficient milk supply [5, 6]. Little is known about the percentage of term parents with a perceived insufficient milk supply who do not produce enough milk (insufficient milk supply) to meet the infant’s demands. Insufficient milk supply is associated with preglandular (metabolic disorders), glandular (chest/breast surgery or malformations), and postglandular (infant and pumping factors, maternal medications/substances, or premature birth) factors [7]. For those factors that may be modifiable, researchers and clinicians need reliable, immediate, and cost-effective strategies to assess milk intake before and after an intervention. Deuterium dilution is a reliable method for measuring milk intake but requires serial measurements and complex equipment. Diaper counts or clinical indicators can identify adequate intake but not changes in milk volume. Multiple studies have used test weights (TWs, weighing an infant before and after feeding) to measure intake and support breastfeeding confidence [8, 9]. However, the accuracy of TWs has only been validated with preterm infants [10–12]. No studies have assessed the accuracy of parents in community settings with term infants and older children. Quantifying milk intake using TWs is immediate, accessible, and noninvasive for clinicians and researchers. However, recent validation studies investigating the accuracy of TWs outside the controlled hospital environment or among term infants and toddlers are limited. This study’s primary aim was to identify how accurate parent infants/toddler TWs are at measuring infant intake with limited investigator involvement. To do this, we first determined the accuracy of milk intake by measuring the change in bottle weight with an infant scale and a more precise digital balance. Next, we compared the milk intake with the change in bottle weight and the parent-infant/toddler TWs. Finally, we accounted for any confounding variables contributing to weight differences or inaccuracies.

## Methods

### Participants

This prospective correlative study compared differences between infant/toddler TWs and human milk bottle TWs between June 5, 2019, and July 30, 2021. The Hope College Human Subjects Review Board approved this study. An addendum was approved in July 2020, implementing strategies to reduce possible COVID-19 transmission. Before participation in any feeding sessions, written informed consent was obtained under the institutional review board requirements.

Individuals were recruited via snowball sampling using social media advertising (Facebook and Instagram), word of mouth, and fliers in infant stores and local community events. Social media posts targeted a 50-mile radius from the research site with the following keywords: community, lactation, babywearing, doula, childbirth, parent groups, milk-sharing, people of color, and indigenous. Individuals older than 18 years, still fed human milk to their infant or toddler, or lived within 35 miles of the research facility were eligible for the study. Their infant or toddler had to be under 33 pounds (due to infant digital scale limitations), greater than 40 weeks corrected gestational age, able to feed human milk from a bottle or cup, and have no untreated gastrointestinal reflux. A $10 incentive was offered and provided to all who completed a feeding session.

To obtain statistical significance, a minimum of 86 feeding sessions was calculated using Rankin and colleagues’ s’ [11] results, assuming a mean difference between the estimated and actual volume of − 1.47 mL (SD = 3.72) using a 95% power and α of 0.05 using G*Power: Statistical Power Analyses for Mac (version 3.1.9.4) [13].

### Measurements

#### Scale/balance

Infant/toddler weights were obtained using a Tanita BD-815U digital scale (0–6 kg/2 g, 6–15 kg/5 g accuracy, 15 kg capacity, Tanita). Bottles were measured using both the Tanita BD-815U digital scale and OHAUS Scout™ SPX222 portable digital balance (± 0.01-g accuracy, 220-g capacity, OHAUS).

#### Demographic characteristics

Maternal and infant characteristics were collected during the initial enrollment using Qualtrics, cloud-based survey management software. Maternal characteristics included age, race/ethnicity, household income, marital status, health insurance type, highest level of education, and employment. Infant characteristics included gestational age at birth, birth type (vaginal or cesarean), sex, age at feeding session, and most frequent feeding method (breast, bottle, or cup). Infant age (months) was calculated using the date of the feeding session and the infant/toddler’s date of birth.

#### Feeding session data

For each feeding session, the research assistant collected the date and time of feeding, recorded the feed duration, and observed any irregularities. An irregularity was any observed change or occurrence during a feeding session that might contribute to a significant variation in infant/toddler weights. The irregularities included an infant length exceeding the length of the scale, excessive infant/toddler movement during the weight, infant/toddler vomiting during the feeding or before the final weight, or an infant/toddler consuming a small amount (< 5 mL) of human milk. Since the research team desired to provide results close to an in-home setting, TW irregularities were not removed from the analysis. Age at the time of the feeding session was calculated by subtracting the feeding session date from the date of birth.

### Variables

#### Bottle test weights

Research assistants obtained the bottle TWs using both the Tanita and OHAUS scales. The average of two pre- and postfeed measurements was calculated. The bottle TW was the mean difference between the post- and prefeed measurements.

#### Infant/toddler test weights

Parents were given verbal instructions on obtaining the infant/toddler TWs using only the Tanita scale. Parents zeroed the scale, placed the infant on the scale until the “weight lock” symbol was on, documented weight, fed the infant, zeroed the scale, and reweighed the infant. The average of the two pre- and postfeed measurements was calculated. The infant/toddler TW was the mean difference between the post- and prefeed measurements.

#### Volume

Volume was calculated by taking the difference in bottle weight before and after the feed and multiplying it by the specific gravity of the milk. Specific gravity was measured using Fisherbrand™ handheld digital clinical refractometer (serum protein ± 0.2; refractive index ± 0.0003nD). Since feeding measurements were collected in grams, only when appropriate measurements are reported in mL based on the average milk specific gravity.

### Protocol

After providing consent, the participants completed a demographic survey and received instructions to sign up for a feeding session with a research team member. Feeding sessions took place at the research institution, community partner institution, or participant’s home within 35 miles of the research institution with a trained research assistant. Participants were permitted to complete more than one feeding session with more than one infant or toddler. The parent was instructed to have ready upon arrival expressed human milk and a bottle the infant had successfully drank from to be weighed by the researcher and fed by the parent. Participants were permitted to use human milk expressed at any time of day, fresh or thawed from frozen milk. Due to the 220-g capacity of the OHAUS digital scale and the large variations in bottle weights (30–190 g), the research team initially predetermined the maximum feed volume to be 30 g, but during the study, volumes were increased to not exceed the digital scale’s capacity.

Before each feeding session, the researcher calibrated the Tanita digital scale and OHAUS digital balance. Before the parent or researcher completed the TWs, the scales were placed on a flat surface (table or floor), leveled, disinfected, and zeroed by the research assistant. The principal investigator (AEZ) oversaw the initial feeding sessions with each researcher to validate the techniques and ensure protocol adherence. During the start of the COVID-19 pandemic (May 2020–July 2021), all the participants were moved to their homes. Research assistants and participants were required to complete COVID-19 screenings guided by the Centers for Disease Control’s recommendations. During feeding sessions, the researcher wore disposable gloves, surgical masks, goggles, and a gown and maintained a minimum distance of 6 feet from the parent and infant/toddler. Participants were required to wear a personal or disposable surgical mask provided by the research team.

During each feeding session, the research assistant documented the bottle weight of the previously collected human milk bottle twice before and after each feeding using both the Tanita and the OHAUS scales. Participants were encouraged to freshly diaper their infant/toddler before the feeding session. After the scale was zeroed, the participants recorded the weight of their infant/toddler twice before and after the infant/toddler feeding. The parent was then instructed to feed the infant the preweighed bottle. The research assistant timed the feeding session and documented any observed irregularities. After the bottle was empty or the infant/toddler refused to drink any milk, the feeding session was stopped. The parent then reweighed the infant in the same fashion as the first weight. Both the researchers and participants remained blinded to the obtained weights during the feeding session.

### Data management

Demographic data from Qualtrics were exported to an Excel spreadsheet and merged with the transcribed feeding session and milk analysis data. The Excel spreadsheet was exported to a CSV file for analysis. Participants received a feeding session report with weight data and milk analysis results after each feeding session.

### Statistical analysis

Agreement between the two bottle TW methods (Tanita and OHAUS scales) was analyzed by calculating the differences between TWs measured using the two methods for the same feeding sessions. Descriptive statistics were computed, including the means and standard deviations (SD), and median and interquartile range 1 and 3 (Q1, Q3) were computed for parametric and non-parametric data, respectfully. Means and SDs were used to estimate 95% limits of agreement (mean difference ± 1.96 SD of differences) for the differences between the two methods. The 95% confidence intervals (CIs) were also constructed for the mean difference and the limits of agreement [14], and a one-sample *t-*test was performed to test the null hypothesis that the true mean difference was equal to zero. A scatter plot (a Bland‒Altman plot) was created to show the differences between the paired measurements plotted against their mean [14]. Finally, the Pearson correlation coefficient quantified the linear correlation between two measurements. All calculations were performed, and all plots were created using R statistical software version 4.1.2. [15].

Analysis of the agreement between bottle TWs (OHAUS) and infant/toddler TWs (Tanita) proceeded in the same manner as comparing the two bottle TW methods. Additionally, a linear regression analysis was conducted. Comparisons were made between four linear models predicting bottle TW in terms of infant/toddler TW and (for one of the models) the duration of the feeding session. The simplest linear model (model 1) predicted that bottle TW was equal to the observed infant/toddler TW. Model 2 was adjusted for a fixed bias between the two measurements (slope equal to 1 but nonzero intercept). Model 3 also allowed for a slope different from 1. Model 4 included both infant/toddler TW and feeding duration as predictors. Thus, model *i* was nested in model *i* + *1* for *i* = 1, 2, 3. Models 2–4 were fit using least squares regression (no parameters were estimated for Model 1). The four models were compared using the residual standard error (RSE), a measure of the average difference between the observed bottle TWs and the bottle TWs predicted by the model (the average prediction error). None of the statistical models included a participant effect due to the lack of replication for most of the participants. Most participants who completed a feeding session completed only one session (17 out of 31).

## Results

### Sample

Of the 57 consenting participants, 31 completed at least one feeding session. Sample characteristics were compared between those who did and did not complete feeding sessions, and no significant differences were noted. The participants were predominantly White (94%, *n* = 33), had private insurance (91%, *n* = 32), and were college graduates (60%, *n* = 21). Additional maternal and infant characteristics of those who completed feeding sessions can be found in Table 1.

**Table 1:** Maternal and infant characteristics of participants who completed a feeding session

**Maternal**	**(*n*** **= 31)**
Age (years), mean ± SD	31 ± 3.6
White/Non-Hispanic, n (%)	33 (94)
Income category per year (median)	$75,000–99,999
Married marital status, n (%)	34 (97)
Private insurance, n (%)	32 (91)
Highest level of education-college graduate, n (%)	21 (60)
Employed full-time, n (%)	14 (40)
**Infant/toddler**	**(*n*** **= 35)**
Gestational age at birth (weeks), mean ± SD	39.1 ± 1.4
Vaginal birth, n (%)	20 (57)
Male infant/toddler sex, n (%)	25 (45)
Primary feeding method of milk- breast, n (%)	30 (86)
Age at feeding session (months), mean ± SD	8.2 (0.3)
Clothed weight at feeding session (kilograms), mean ± SD	7.7 (2.1)

#### Feeding session data

Thirty-one participants completed 101 feeding sessions. Feeding sessions took place at the research institution (*n* = 12), community partner institution (*n* = 8), or participant home (*n* = 81). The average duration of the feeding session was 4.8 min (SD = 4.5). The average bottle weight prefeed per OHAUS scale was 137 g (SD = 55.6). The average specific gravity of the human milk was 1.034 (SD = 0.004, Min, Max: 1.027, 1.050), with a median volume consumed of 32 ml (Q1, 24.8, Q3 55.8). The researcher assistants documented 21 (21%) cases of TW irregularities, with 11 of the occurrences being related to excessive infant movement.

#### Tanita and OHUAS bottle test weights

The results comparing the two bottle TW methods are summarized in Table 2.

**Table 2:** Limits of agreements of test weights

	**Difference (g)**				**Limits of Agreements (g)**		**CI of Limits of Agreement (g)**		
	**Range**	**Mean**	**SD**	**CI**	**Lower**	**Upper**	**Lower**	**Upper**	**r**
Tanita vs. OHAUS Test Weight	4.49	-0.0718	0.896	(-0.251, 0.108)	-1.83	1.68	(-2.14, -1.51)	(1.38, 1.99)	0.9996***
Bottle vs. Infant/toddler Test Weight	45.5	-2.18***	6.33	(-3.45, -0.915)	-14.6	10.2	(-16.8, -12.4)	(8.04, 12.4)	0.9795***

Range: Difference between the lowest and highest values

*SD* Standard Deviation, *CI*: 95% Confidence Interval

*** indicates *p* < 0.001

**Fig. 1:**
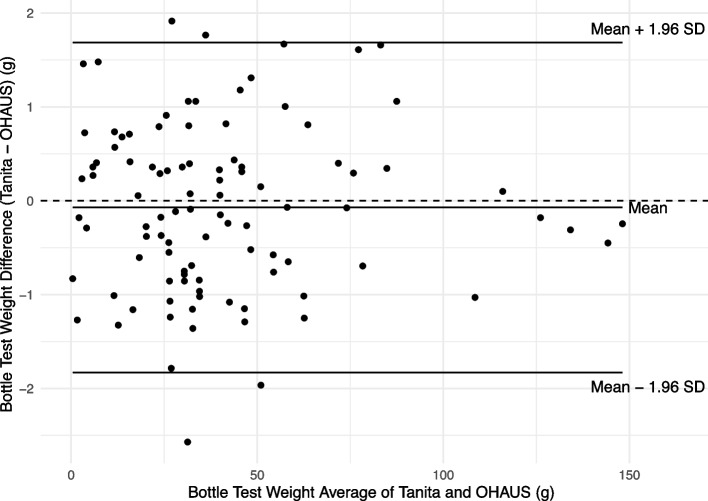
Bland–Altman Plot showing the mean differences and limits of agreement of Bottle Test Weights using Tanita and OHAUS scales

The Tanita and OHAUS bottle TW measurements were strongly correlated (*r* = 0.9996, *p* < 0.001). The Bland‒Altman plot in Fig. 1 shows the agreement between the two methods using the Tanita and OHAUS scales.

The mean difference between the measurements was not significantly different from zero (mean difference (Tanita—OHAUS) = -0.072 g,* t* = -0.793, *p* = 0.430). This indicates that there is no significant fixed bias between the two methods. There was no apparent variation in either the mean or standard deviation of the differences over the range of the measurements (Fig. 1). The 95% limits of agreement are -1.83 to 1.68 g, indicating that we expect approximately 95% of the differences between measurements using the two methods to fall within these limits. The confidence intervals for the limits of agreement are given in Table 2.

#### Bottle and infant/toddler test weights

**Fig. 2:**
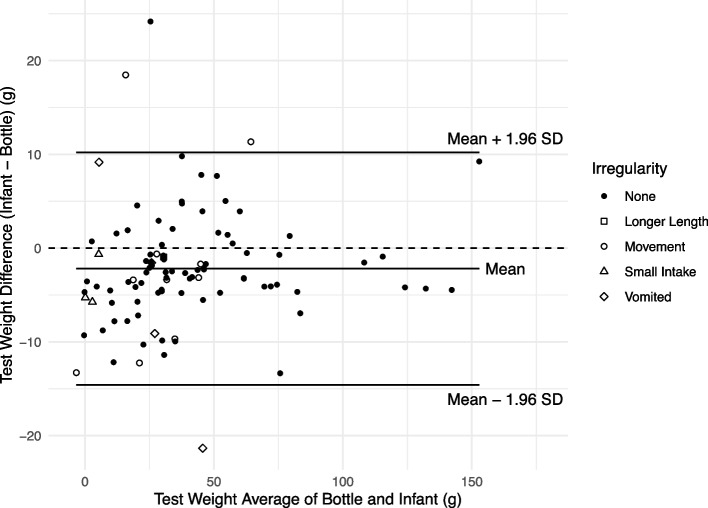
Bland Altman Plot showing the mean differences and limits of agreement of Infant/toddler and Bottle Test Weights using the Tanita scale. Legend: • None, ☐ Longer Length, 〇 Movement, ∆ Small Intake, ◊ Vomited

The results of the comparison of the infant/toddler and bottle TW methods are summarized in Table 2. The infant/toddler and bottle TW measurements were strongly correlated (*r* = 0.979, *p* < 0.001). The Bland‒Altman plot in Fig. 2 shows the agreement between the infant/toddler and bottle TW methods.

The mean difference between the measurements was statistically significantly different than zero (mean difference (infant/toddler—bottle) = -2.18 g, *t* = -3.42, *p* < 0.001). This indicates that there is a significant fixed bias between the two methods. The parent-measured infant/toddler TWs were, on average, 2.18 g (SD = 6.33) or 2.25 mL lower than the researcher-measured TWs. There is no evidence that this bias or that the standard deviation of the differences varied over the range of measurements (Fig. 2). The 95% limits of agreement are -14.6 to 10.2 g (-15.1 to 10.5 mL), indicating that we expect approximately 95% of the differences between infant/toddler and bottle TWs for the same feeding session to be between these limits. The confidence intervals for the limits of agreement are given in Table 2. Figure 2 highlights the infant/toddler TW irregularity types by symbol. Figure 2 shows that vomiting and movement contributed to the greatest irregularities (three of the four differences falling outside the limits of agreement), while differences were inside the limits of agreement for most movements, longer lengths, or small intakes. Excluding irregularities somewhat narrows the limits of agreement, but they are retained in the analyses presented here.

### Predicting bottle test weights

The feeding session duration was linearly associated with the bottle TW after adjusting for the infant/toddler TW (*p* = 0.028). The linear model predicting researcher-measured bottle TWs (the best estimate of the mass of human milk consumed during the feeding session) in terms of parent-measured infant/toddler TWs is listed in Table 3 (Model 4). The feeding session duration and infant/toddler TW predictors were correlated (*r* = 0.328, *p* = 0.006).

**Table 3:** Linear models for predicting researcher-measured bottle test weight

Model	Fitted model	RSE	
1	*Predicted Bottle TW* = *Infant/Toddler TW*	6.66 g	6.89 mL
2	*Predicted Bottle TW* = *1.99* + *Infant/Toddler TW*	6.33 g	6.55 mL
3	*Predicted Bottle TW* = *3.80* + *0.952* × *Infant/Toddler TW*	6.22 g	6.43 mL
4	*Predicted Bottle TW* = *2.50* + *0.926* × *Infant/Toddler TW* + *0.505* × *Duration*	6.08 g	6.29 mL

*RSE* residual standard error, *TW* test weight

**Fig. 3:**
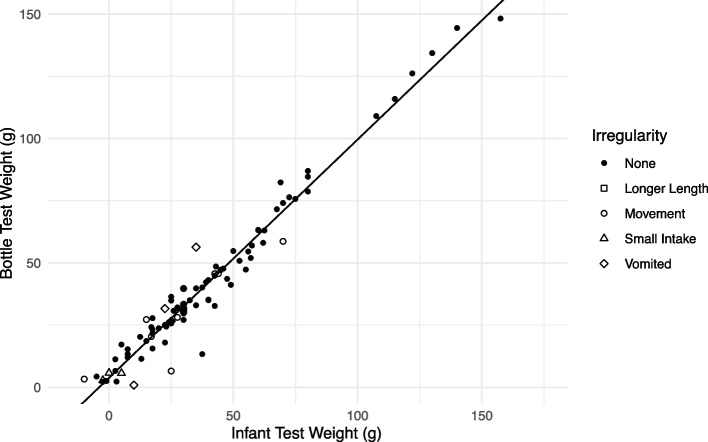
Regression Model 3: Bottle Test Weight vs. Infant/Toddler Test Weight. Legend: • None, ☐ Longer Length, 〇 Movement, ∆ Small Intake, ◊ Vomited

Researcher-measured bottle TW is plotted against parent-measured infant/toddler TW in Fig. 3.

The figure also shows the least squares regression line for Model 3. Most points with large residuals are associated with infant/toddler TW irregularities (indicated by symbols in Fig. 3). The plot shows a strong linear relationship between infant/toddler and bottle TWs (*r* = 0.979, *R*^2^ = 0.959, *p* < 0.001). The accuracy of all four linear models was quantified using the RSE (Table 3). The accuracy improved as more complicated models were considered, decreasing from 6.66 g (6.89 mL) for the simplest model (Model 1) to 6.08 g (6.29 mL) for the model that incorporated infant/toddler TWs and feeding session duration as predictors (Model 4).

## Discussion

Our analysis of the comparison of human milk bottles with infant/toddler TWs is the first known published study exploring the accuracy of infant/toddler TWs by a parent in the community and home setting using portable digital infant scales. Our data demonstrated that the Tanita infant scale accurately measured the change in milk weight compared to the more precise OHAUS bottle scale, as both scales obtained the same milk weight. Despite a statistically significant difference between infant/toddler TWs using the Tanita TWs and bottle TWs using the OHAUS TWs, the mean difference was minimal (2.18 g or 2.25 mL) and within an acceptable range with our population (7.7 kg weight average), similar to the manufacturer’s parameter of less than 2 g for infants weighing less than 6 kg and 5 g for toddlers weighing 6–15 kg. This difference is small enough not to affect the monitoring of milk intake and overall infant health and growth. Our predictive modeling identified that the difference was not dependent on the volume of milk consumed. Therefore, when parents use TWs to measure intake, the scales perform according to their specifications, even with larger infants and toddlers. This can be valuable to both parents and clinicians to reassure them that the infant intake is enough and reduce early lactation discontinuation due to perceived insufficient milk supply.

Our analysis predicted infant intake (bottle TW) with an RSE range of 6.08–6.66 g (6.29–6.89 mL). Since RSE measures the average prediction error (the average size of the difference between the predicted and observed bottle TWs), smaller RSE values indicate higher model accuracy. Because RSE considers the model’s degrees of freedom, a decrease in RSE with increasing complexity of nested models is not guaranteed. However, this reduction in prediction error with increasing model complexity (0.58 g between Model 1 and Model 4) is not clinically significant. Therefore, we recommend using Model 1, which predicts bottle TWs as equal to infant/toddler TWs, or Model 2 (addition of 2.18 g or 2.25 mL to infant/toddler TWs), which adjusts for a small fixed bias. One limitation of the analysis is that the statistical models did not account for participant effects due to a lack of replication for most participants.

Our findings differed significantly from those of Savenije and Brand [16], who weighed 100 hospitalized infants 15 min after the infant feeding was completed. The primary differences between our study and that of Savenije and Brand were the age and size of the infants, the complications of feeding tubes and monitoring cables, and the duration between feeding and weight. We were not without our complications, as we had irregularities such as excessive infant/toddler movement (11 cases) and vomiting (3 cases). However, Figs. 2 and 3 both visualize that the incidents of excessive movement did not significantly contribute to the intake variation. Even though preventing infant vomiting may be difficult, we speculate that most of the excessive movement was related to the infant/toddler only being fed one ounce of human milk. We presume that when infants are fully satiated that excessive movement would be minimal.

The difference identified between the bottle and infant/toddler TW, or the positive coefficient for feeding, is consistent with what we expected because infant/toddler mass decreases through insensible or evaporative water loss during feedings [17]. We expected the infant/toddler TW to underestimate the bottle TW and that the difference would increase with the duration of the feeding session. Since the feeding session duration and infant/toddler TW predictors are correlated, clinicians and researchers should take care in interpreting the precise values of the slopes in this model. Our findings are not unique. Butte and colleagues reported a 3.0 ± 2% difference in bottle and infant weights [18]. While Borschel and colleagues reported that infant weight underestimated infant intake by 4–9%, one major difference in our study was using digital scales designed to measure breastfeeding infant intake [19].

### Limitations

Limitations affecting this study were related to the COVID-19 pandemic, sample size, and feed volume and duration. Initially, we had a high interest in participation. However, because of the pandemic, few parents of young infants (< 3 months) were willing to have a research assistant in their home, resulting in older infants and some participants completing multiple feeding sessions. For those enrolled who completed multiple feeding sessions, our analysis did not find that more feeding sessions resulted in lower TW differences between the bottle and infant/toddler. The location of the study site and the pandemic also limited our sample socioeconomic and educational diversity since few (16%) low-income minority women in the Special Supplemental Nutrition Program for Women, Infants, and Children are breastfeeding at all at six months in this region [20], and diverse community health leaders recommended limiting COVID-19 exposure; we were unable to enroll a diverse population. Additionally, we decided early on that parents should feed their infants in their preferred location (home, community setting, or research lab) using their preferred infant/toddler bottle. Due to the large range of bottle weights and weight limitations of the digital balance, our bottle volumes were initially limited to 30 mL. Nevertheless, our analysis corroborated that the weight differences were not volume dependent, and the feeding irregularities did not significantly contribute to the difference but would have narrowed the limits of agreement. In addition to our small volumes, our infants consumed the milk quickly. Since we have identified the accuracy of the Tanita scale, additional studies could be conducted with larger volumes until infants are satiated.

## Conclusions

The Tanita infant digital scale accurately measures milk intake, and parents can reliably measure infant intake for older infants/toddlers. Since most infant intake concerns occur after hospital discharge, lactation providers and community health workers can rely on parents in their home setting with minimal instruction to measure their infant intake. Furthermore, researchers can use parents to accurately and reliably measure the immediate impact of interventions developed to increase milk volume.

## Data Availability

Individuals interested in accessing the data from this study can contact the corresponding author.

## Abbreviation

TW: Test Weight
